# Overcoming Challenges to Treating Tobacco use During Pregnancy - A Qualitative study of Australian General Practitioners Barriers

**DOI:** 10.1186/s12884-019-2208-8

**Published:** 2019-02-07

**Authors:** Yael Bar-Zeev, Eliza Skelton, Billie Bonevski, Maree Gruppetta, Gillian S. Gould

**Affiliations:** 1 Centre for Brain & Mental Health Research, University of Newcastle, Callaghan, 2308 Australia; 2 The Wollotuka Institute, University of Newcastle, Callaghan, 2308 Australia

**Keywords:** Smoking cessation, Pregnancy, Health providers, nicotine replacement therapy

## Abstract

**Background:**

General practitioners can play an important role in addressing smoking among pregnant women but studies suggest they rarely do so. The aim of this study was to explore general practitioners perceptions about the management of smoking in pregnancy, and what would enable them to provide better care.

**Methods:**

Qualitative semi-structured interviews were conducted (Feb-July 2017), with 19 Australian general practitioners recruited from a sample that participated in a national survey on managing smoking during pregnancy; and through a national conference. The interview guide was structured using the theoretical domains framework, exploring previously reported barriers and two specific components of smoking cessation care - nicotine replacement therapy prescription and Quitline referral.

**Results:**

Participants reported high confidence and knowledge to provide pregnant patients adequate support for quitting. Nonetheless, participants reported lacking communication skills, focusing on providing information on smoking harm, accepting cutting down cigarettes as adequate, while following the ‘Stages of Change’ model and only providing treatment options to motivated patients. Lack of time, nicotine replacement therapy cost and safety concerns, and being unfamiliar with the Quitline (particularly for Aboriginal and Torres Strait Islander pregnant smokers) were perceived as challenges. Participants reported needing better communication skills, clear detailed nicotine replacement therapy guidelines for special populations, and visual resources they could use to discuss treatment options with patients.

**Conclusions:**

Difficulty communicating with pregnant patients about smoking, using the ‘Stages of Change’ model to guide support provision and concerns regarding nicotine replacement therapy safety are barriers to providing cessation support to pregnant patients for general practitioners. Training on specific effective behaviour change techniques, clear guidance for nicotine replacement therapy use, and practical visual patient education tools may facilitate smoking cessation care provision to pregnant women.

**Electronic supplementary material:**

The online version of this article (10.1186/s12884-019-2208-8) contains supplementary material, which is available to authorized users.

## Background

Smoking during pregnancy remains a global public health challenge [1, 2], and is an important risk factor for poor maternal and infant health outcomes [3]. Globally, rates of smoking among pregnant women range from 7 to 18% [1]. In Australia, in 2016, 9.9% of pregnant women reported smoking. Higher smoking rates are found in younger mothers under the age of 20 years (30.5%), living in the lowest socio-economic areas (17.4%) and Aboriginal and Torres Strait Islander women (42%) (hereafter referred to “Aboriginal” women with acknowledgement of the distinct cultures) [4].

Internationally recognised clinical guidelines recommend using the 5A’s approach when treating pregnant women who smoke [5]. The Royal Australian Collage of General Practitioners (RACGP) also recommend using the 5A’s [6] and structure the recommended counselling approach using the Trans-Theoretical theory, i.e. the ‘stages of change’ model [7]. According to this theory, smokers transition though a cycle of readiness to change their behaviour [7]. Hence RACGP guidelines recommend assessing the patients’ motivation to quit, and tailoring advice accordingly [6]. However, evidence now suggests that this approach is outdated, and interventions based on stages of change have not been shown to be more effective than non-stage-based interventions [8].

Additionally, these guidelines recommend initially only behavioural counselling, but if this is unsuccessful, the pregnant woman should be offered nicotine replacement therapy (NRT) after weighing the risks versus benefits [6]. A meta-analysis of studies indicate that NRT combined with behavioural support might increase cessation rates by 40% [9]. Nicotine in itself has been found in animal studies to be harmful for the foetus brain and lung development [3, 10], but studies in humans have not found any evidence of harm [9, 11]. Therefore, expert opinion in Australia and other countries is that NRT is always safer than continuing smoking [6, 11–13]. The RACGP guidelines recommend oral NRT as first line pharmacotherapy, then NRT patch followed by combination NRT (oral plus patch) [6].

Previous research has shown that globally health providers are not providing adequate smoking cessation care (SCC) during pregnancy. Studies report low rates of assisting pregnant women to quit, referring to other cessation support, including the Quitline, and prescribing NRT [14–16]. Multiple barriers have been identified in the past including lack of knowledge and skills, lack of confidence in ability to counsel and prescribe NRT, lack of time and resources, perceptions that patients do not want to be advised, and doing so would be detrimental to the provider-patient relationship [14, 15, 17].

A recent Australian national cross-sectional survey with 378 general practitioners (GPs) and obstetricians, found similar results to other international studies [18, 19]. A high proportion of clinicians reported always ‘Asking’ (77%) and ‘Advising’ to quit (75%), but lower proportions reported always doing the ‘Assess’ (24%), ‘Assist’ (33%), and ‘Arrange’ (7%) components [18]. Furthermore, 25% stated they would never prescribe NRT, and over 50% had some concerns regarding NRT safety [19]. Only 26% stated they always refer pregnant patients to the Quitline. The Theoretical Domains Framework (TDF) is a validated and integrative theoretical framework that covers a range of domains relevant to professional practices and behaviour change [20]. Using the TDF revealed that the most frequently reported barriers were lack of time and resources, lack of optimism, and lack of confidence in their ability to prescribe NRT [18, 19].

To date, very few qualitative studies have been conducted to fully understand these barriers and what would facilitate health providers to better manage smoking during pregnancy [21]. In Australia, only two studies have been conducted so far, and neither included GPs [22, 23]. The purpose of this study was to explore the perceptions and attitudes of GPs consulting with pregnant women who smoke, and what would enable them to better manage smoking in pregnancy. The study aims to describe their individual experiences with providing SCC to pregnant women who smoke and what would facilitate them to overcome known barriers.

## Methods

### Participants and recruitment

Participating GPs (*n* = 19) were recruited from two samples:

1. 118 GPs were invited from a sample that took part in the national survey mentioned above [18, 19, 24] and gave consent to be further contacted. This database also provided the participants’ socio-demographic data and self-reported knowledge, attitudes and actual practices.
2. During the 2016 National Australian GPs conference, the study was advertised. Interested GPs were asked to contact the research team for further information (*n* = 4 responded).

An email invitation was sent to all 122 GPs, and a reminder email was sent to those that didn’t reply. The email included a detailed information sheet, outlining the purpose of this study. In addition, purposive sampling was conducted using a third separate email to try to sample GPs who had reported both high and low levels of SCC provision in the survey. However, no low level care providers were recruited. We included in the study all of the GPs who responded to our invitation, resulting in 16 GPs recruited through the survey database and 3 GPs recruited through the conference.

### Procedure

Telephone interviews were conducted between February to July 2017, by one researcher (YBZ) using a semi-structured interview guide (Additional file 1). The interview guide was structured using TDF [20] domains that were reported in the national survey as barriers [18, 19]: Environmental Context and Resources (lack of time and resources); Beliefs about Capabilities (Confidence in prescribing NRT); and Optimism. Two components of SCC were specifically explored – NRT prescription, and Quitline referral. The interview guide was not pilot tested. At the beginning of the interview, the researcher introduced her personal background and reasons for conducting this study, explained the aims of the study, gained and recorded the participants’ verbal consent. YBZ is a female Public Health Physician and Tobacco Treatment Specialist, with extensive experience in training physicians regarding smoking cessation. This study was done as part of her PhD, and prior to conducting this study, she received specific training on qualitative data collection and analysis. The study was approved by the University of Newcastle Human Research Ethics Committee (08/06/2016, H-2016-0063).

### Analysis

Transcription was completed by a professional service. Interviews were read repeatedly and then coded line by line using an inductive general thematic approach [25], with NVivo software (version 11). Initially, a subset of the data (*n* = 5 interviews) was independently coded by two researchers (YBZ and ES), and a coding manual developed. The coding manual was used by one researcher (YBZ) to code the remaining transcripts. If new themes were interpreted, they were discussed and agreed upon with the second coder (ES). ES is a female health behaviour scientist with prior experience in qualitative analysis. This process enabled researcher triangulation, reducing bias, and enhancing transferability and confirmability of the findings. In addition, field notes were kept during the data collection process, to capture the researcher’s thoughts, opinions and feelings, and were reflected upon during the analysis. In particular, the researcher was aware during the data collection and analysis that her own background as a tobacco treatment specialist might bias her interpretation of physicians’ experiences, and reflected upon this using the field notes.

## Results

Reporting was guided by the COREQ checklist [26].

### Participant’s characteristics

Participants came from all Australian states, except the Australian Capital Territory. Socio-demographic characteristics are detailed in Table 1. Interview length was, on average, 26 min (range 18–46). Data regarding self-reported practices and attitudes from the 16 survey participants are provided in Additional file 2.

**Table 1:** sociodemographic characteristics of participants and medical practice settings

Variable	*N* (%)
Gender - Female	16 (84.2%)
Age (missing *n* = 4)	
*Under 44*	6 (42.9%)
*45–60 years old*	6 (42.9%)
*Over 60 years*	2 (14.3%)
Obstetric training (missing *n* = 3)	13 (81.3%)
Years since medical qualification (missing n = 3)	
*< 10 years*	4 (25%)
*10–19 years*	5 (31.3%)
*20 plus years*	7 (43.8%)
Smoking status (missing *n* = 3)	
*Ex-smoker*	2 (12.5%)
*Never smoker*	14 (87.5%)
Medical Practice	
*Urban*	6 (31.6%)
*Regional*	10 (52.6%)
*Remote*	3 (15.8%)
Population usually caters for (missing *n* = 1)	
*General population*	10 (55.6%)
*Over 30% Aboriginal and Torres Strait Islander*	8 (44.4%)
State	
*New South Wales*	3 (15.8%)
*Queensland*	4 (21.1%)
*Victoria*	4 (21.1%)
*South Australia*	1 (5.3%)
*West Australia*	3 (15.8%)
*Tasmania*	1 (5.3%)
*Northern Territory*	3 (15.8%)
*Australian Capital Territory*	0 (0%)

### Themes

#### Mixed feelings regarding managing smoking during pregnancy

Participants demonstrated a mixture of feelings about optimism. Some were somewhat optimistic, mainly due to perceiving women as more receptive to change due to the pregnancy *“Probably optimistic because they do have that added incentive to quit, that sometimes it’s a really good opportunity to get them to quit.” (#10, Female, under 30, Tasmania)*. Others were pessimistic, mainly due to recurring cases of continuing smoking, and related to all the other psychosocial issues that were out of their abilities to care for *“I suppose I feel defeated by the people’s condition, too pessimistic about the people’s condition. So much needs to change in terms of changing tobacco.”(#1, Female, over 60, Northern Territory); “I have had so many experiences where I feel like I’ve provided a lot of education and time I’ve spent invested in trying to help the pregnant woman understand how harmful smoking is and yet she continues to smoke. I think that’s disheartening when you see the effects and you know you have tackled the problem and continue to address it, but that doesn’t necessarily change the patient”(#5, Female, Queensland, age unknown).*

Participants viewed addressing smoking as an important part of their role, and viewed their relationship with the patient as imperative to reaching the patient *“there is benefit of having us there… as their regular health professional. I think it does make a huge difference to how much they’re likely to listen to that advice and take it on board.” (#5, Female, Queensland, age unknown).* Nonetheless, it was evident that they felt that combating smoking in pregnancy is not just a medical condition they can treat, and would require other policy measures that address the psychosocial factors that also impact smoking, and make quitting more difficult *“when you’ve got overcrowding, domestic violence, you were abused as a kid, be it physically, emotionally or sexually, when there’s flies crawling all around the floor, when everybody else in the house smokes, I just feel like it’s just such a mountain.” (#1, Female, over 60, Northern Territory).*

Time was perceived as problematic for some, usually in relation to other competing priorities. *“…something else would have to get cut out. There isn’t really anything you can cut out is the problem…” (#4, Female, 31–44, West Australia).* Others, especially those working within Aboriginal Medical Services, found this was not an issue *“Fortunately, we’re not as time bound as a city general practice” (#1, Female, over 60, Northern Territory).*

#### Current practices were suboptimal

When asked about their approach to managing smoking, participants described using the “stages of change” approach [7] *“GP guidelines for quitting have got the whole ‘stages of change’….” (#10, Female, under 30, Tasmania).* Women who were perceived as ‘not ready’ were provided with information on smoking harms; whereas women who were perceived as ‘ready’ to quit, were offered options for support *“I give them the information that they needed in order to make a decision, so make sure they knew about the harmful effects of smoking and determine their level of motivation and confidence in quitting, and if they were ready to quit, then we talk about the different ways of doing so.” (#11, Female, 45–60 years, West Australia).* Participants also stated they would not mention the Quitline if they felt patients were not ready to quit *“If people don’t indicate to me that they’re interested in a planned cessation or decreasing, I don’t refer them to the Quitline.” (#12, Female, over 60, New South Wales)*. Participants emphasized a strong focus on providing information on the harms of tobacco smoking: *“explaining to them about the risks of pregnancy, explaining that it is extremely harmful… to understand that the smoking that they’re doing is harming their baby” (#5, Female, Queensland, age unknown).* Most participants accepted cutting down consumption as an adequate method for managing smoking during pregnancy *“for the person who says ‘Well I’ll just smoke the minimum and that’s the best I can do’, I accept that.” (#2, Male, 45–60 years, Victoria).*

#### Needing better communication skills

Participants expressed a need to learn ‘how’ to have conversations to support women in their quit journey. They wanted this shown to them explicitly (as opposed to just providing information): *“I don’t feel like I know that very well because we don’t really learn that in med school. We learn a lot of the medical issues with smoking, but we’re not learning the psychology of smoking. It could even be just we watch a DVD and watch someone pattern a role model.” (#4, Female, 31–44 years, West Australia)*.

It was important to the participants to maintain a positive relationship with the pregnant patient *“there’s real caution in when to push it and when to slack off a little bit and don’t say anything, but it just means you don’t make the person feel guilty and they’ll never want to see you again and you lose your influence altogether.”(#6, Male, Queensland, age unknown).*This led participants to be wary of the way they were conveying the message “*I’m inclined to just kind of put my blinkers on, I sort of bite my tongue a little bit when I know that it’s going to make the patient upset, or angry… it’s a tough issue, really tricky.” (#19, Female, 31–44 years, Northern Territory)*. Furthermore, participants talked about trying to provide information in a non-judgmental and supportive way *“it is a delicate conversation to be had with the patient because you are telling them that what they are doing is potentially harming their baby, people can get very defensive, you want to maintain that rapport and you don’t want to be judgmental,” (#14, Female, 31–44 years, New South Wales).* Acceptance of ‘cutting down’ was related to wanting to maintain good rapport and being supportive *“I congratulate them on cutting down. She knows that she’s not doing the best by her baby or by herself, so forcing the issue and making her feel more bad about herself than she already is, it’s counter-productive”(#6, Male, Queensland, age unknown).*

#### Barriers for NRT prescription

The common experience among participants was that most pregnant women simply did not want to use NRT *“There’s quite a number of women who just aren’t interested… even in spite of reassurances that nicotine replacement is preferable to smoking… will say ‘No thanks. That’s just not quite me.” (#9, Female, 41–60 years, Queensland).* This was related to safety concerns *“They feel that their baby would be better off if they were to smoke intermittently rather than have constant nicotine” (#2, Male, 45–60 years, Victoria);* or to women’s negative experiences from prior use *“Women are afraid about using patches and then the other half have used them before when they weren’t pregnant and refused to use them again.” (#7, Female, New South Wales, age unknown).*

Most of the participants were comfortable prescribing NRT during pregnancy (also evident in their survey answers, Additional file 2), and stated that NRT was safer than smoking. Despite this, some reported concerns *“I always feel a bit concerned about doing actually more harm than good insofar as you know these women that appear to not be smoking very much” (#3, Female, 31–44, Northern Territory)*. Several participants felt that not all pregnant women were physically addicted to nicotine, and that their smoking was due to other reasons such as stress. Therefore, they did not think NRT to be an appropriate treatment in this context. They described NRT as only appropriate to consider in highly addicted smokers, and/or that combination treatment is not suitable *“it depends why she says she’s smoking. If there’s an element of addiction to it… I do suggest they go on patches” (#4, Female, 31–44, Western Australia).*

Participants described several possible facilitators that might help them to improve their confidence and skills regarding NRT prescription:

1. Needing clear and detailed information

Participants expressed a need for clear guidance on when it was appropriate to initiate NRT and how to determine the dose *“I suppose we need sort of like training modules… like an algorithm about ‘This is what you use. This is how you start it. These are the benefits’.”(#8, Female, South Australia, age unknown);* the lack of clear guidance impacted their confidence in discussing NRT “*I would have to look up doses…it might make me a little bit less happy to engage and have a longer consult because I just don’t feel confident with my level of knowledge” (#8, Female, South Australia, age unknown)*.

2) Requiring visual resources

Participants wanted resources to guide the conversation on NRT safety, helping them feel more confident to recommend it in pregnancy, and provide an objective portray for the women *"A very simple kind of handout or even if it’s like a poster in the room… it’s more just as a back-up thing. So, like, “Hey, it’s not just me saying it” (#14, Female, 31–44 years, New South Wales).* Those working with Aboriginal women emphasized the need for a visual culturally responsive resource *“Handouts that are appropriate for my patients, Aboriginal and Torres Strait Islander women… as you’re explaining it, you’ve got these visuals to point to.” (#14, Female, 31–44, New South Wales).*

3) Reducing NRT cost

Those working with Aboriginal people (eligible to receive the patch for free as part of the Australian Government’s Pharmaceuticals Benefit Scheme) viewed the patch as their main option due to the cost of oral NRT, with having the patch at the service for free a major facilitator *“in an Indigenous community, if anything costs money… that’s almost out of the question.” (#19, Female, 31–44 years, Northern Territory).*

#### Barriers for Quitline referral

Participants were aware of the Quitline and have referred pregnant women to it, but most remarked about not being familiar with its process *“it’s sort of like an unknown…I don’t know what happens when people call up to the Quitline, I don’t know if they would get the same counsellor each time or whether they just call up and then get a random person” (#14, Female, 31–44, New South Wales);* feeling disconnected from the treatment their patients were receiving *“.. I’ve been referring to the Quit Line, or giving the numbers to patients for the Quit Line for a long time. I’ve never received any information back and neither have I had any patients tell me that they’ve used it or found it effective.” (#7, Female, New South Wales, age unknown).*

Several participants, specifically those working with Aboriginal women, remarked on the Quitline not being suitable, preventing them from being more proactive *“It wouldn’t be something we’d jump into because of that kind of language and cultural barrier…” (#1, Female, over 60, Northern Territory); “I think it’s pretty unlikely that a young remote Indigenous girl’s going to call the Quitline. I wouldn’t avoid talking about it, but I guess it’s not usually sort of top of my list of things to talk to her about.”(#19, Female, 31–44 years, Northern Territory).* When asked directly, participants working with Aboriginal people did not know that you can request an Aboriginal counsellor *“if you had an Aboriginal Quitline, they might be more likely to use that…. I’ve never actually rung them up and found out.” (#1, Female, over 60, Northern Territory).*

## Discussion

### Main findings

In this qualitative study with Australian GPs from diverse settings, participants reported focusing on providing information on smoking harms, and lacking practical communication skills. Their knowledge is out of date (through concordant with current Australian GP guidelines), still following the ‘stages of change’ model. For some GPs multiple additional challenges are present. These challenges include lack of time, patients’ previous negative experiences about NRT and related safety concerns, and not receiving feedback from the Quitline. Specifically for participants caring for Aboriginal patients, also the cost of Oral NRT, and the Quitline suitability for Aboriginal smokers. Participants were pessimistic about whether they made any difference – their experience is that women continue to smoke despite their efforts to help. Subsequently, this causes participating GPs to be passive, accepting that women may only cut down rather than quit, and offering treatment options only to those whom they perceive as ‘ready’ to quit. In order to overcome these challenges, participants requested practical interactive communication training via ‘role model’ demonstrations, coupled with visual resources, and detailed clear guidelines on the initiation and dosage of NRT.

### Comparison with the literature

Similar to this study, a recent systematic review, synthesizing data from eight qualitative studies from high income countries, highlighted that there is a need for health providers to find better ways to discuss smoking without feeling that this will damage their relationship with the women [21]. Additionally, it was recognized by the health providers that there is a need for smoking cessation support to also be addressed outside the healthcare system, in the broader social context [21]. This review included only one Australian study with seven midwifes [21]. Another Australian qualitative study analysed interviews with 27 maternity service managers, obstetricians and midwives, and reported similar barriers and enablers to those found in our study (lack of knowledge, skills, training), including fear that these conversations would be “difficult” and might damage their relationship with the women [22]. This study did not include any GPs and only included participants from one Australian state (New South Wales) [22].

The uncertainty about the Quitline having Aboriginal counsellors was unexpected. In a small survey with 34 health providers working inside an Aboriginal health service, all of the participants knew this, and most of them found the Aboriginal Quitline counsellors helpful and appropriate [27]. However, this was a small study with most of the participants having received prior smoking cessation training [27].

Concerns over safety of NRT use in pregnancy, and lack of confidence in prescribing it, have been found in numerous studies, from different countries [28–31]. However, all of these were cross-sectional surveys. To date, and to the best of our knowledge, only three qualitative studies explored this issue (from the UK and Canada), and their findings also emphasized the need for clear guidance on NRT safety and prescribing information [17, 32, 33].

### Strengths and limitations

This is the first Australian study, as far as we are aware, to qualitatively explore GPs’ needs in overcoming barriers to providing SCC during pregnancy. Previous qualitative research did not include GPs [21, 22]. As GPs are arguably one of the most influential health providers in primary care, and often provide shared antenatal care, it is important to understand their needs. This is especially true for GPs working with Aboriginal women, as they face additional barriers that need addressing. The fact that participating GPs were recruited from almost all Australian states, and from diverse clinical and geographical settings is an additional strength. The preponderance of female participants may have indicated a respondent bias.

Given that this sample was recruited mainly among participants from a national survey, the previous limitation of the surveys’ low response rate apply to this study and impact the findings transferability. Despite purposely trying to sample low level care GPs, the recruited sample reflected GPs with potentially a higher performance level, and more positive attitudes, compared to the overall national survey results [18, 19] (Additional file 2: Tables S1 & S2). This was a key limitation as we could not explore the needs for overcoming the challenges with those that perhaps have the highest need for improvement. None-the-less, even with these better performing GPs (by self-report), there was still a necessity to improve their knowledge and skills. In fact, this study highlights that even the “best performing” GPs reported many barriers to overcome to provide evidence based SCC for pregnant women.

### Implications for policy and practice

The Trans-Theoretical “stages of change” theory is one of the most well-known behaviour change theories [34]. Despite this, systematic reviews and meta-analyses of studies using stage-based approaches have not shown that interventions designed according to this theory have higher smoking cessation rates than interventions that are non-stage-based [8, 34, 35]. In fact, recent data suggests that the level of motivation to quit is highly variable, possibly fluctuating day to day [36]. This is also supported by the finding that 72% of quit attempts are reported as spontaneous [36]. This study reveals that all of the 19 participating GPs use the ‘stages of change’ model to guide their intervention with pregnant women. Thus if women were perceived to be ‘not ready’, they may not receive valuable information on available smoking cessation support options or assistance to quit smoking. Simply knowing about these options and an offer of assistance might change their level of motivation to quit [37]. Specific training regarding the importance of offering all smokers current cessation options, regardless of their motivation or readiness to quit, is crucial. The New Zealand smoking cessation guidelines [38], recommend an ABC approach (Ask about smoking status, Brief advice to quit. Cessation support): this may be more suitable than the 5A’s currently used in Australia to highlight the need to offer support to all, regardless of their current motivation to quit. The recently developed COM-B model (Capability, Opportunity, Motivation – Behaviour) [39] for behaviour change may be a more suitable model for training GPs. The COM-B model includes additional aspects, other than motivation, that can directly lead to behaviour change – such as improving physical opportunity to women (for example by health providers offering them NRT) [39].

Increasing GPs’ prescribing rates of NRT during pregnancy might increase smoking cessation rates [9]. GPs requested clear practical guidelines including how to make decisions on NRT dosage initiation and titration. Furthermore, patient material that clearly depicts that NRT is safer than smoking and can be used during pregnancy could help guide the doctor-patient discussion on the risk versus benefit. A recent narrative review looking at national clinical guidelines from English-speaking high income countries, including Australia, show that guidelines pose many restrictions on NRT prescribing (for example recommending NRT only for highly motivated pregnant women), and none offered practical details [11]. In an era of overwhelming volume of new data being published every day, clinical guidelines need to provide regularly updated and practical detailed recommendations. Nonetheless, it should be acknowledged that even having regularly up-dated guidelines with practical recommendations does not guarantee this will influence practice by itself.

The barriers to Quitline referral mentioned by the GPs, suggest that familiarizing the GPs with the Quitline process, including the availability of Aboriginal counsellors, may improve referral rates. In addition, finding the means to connect the GP to the Quitline counsellor, and exchanging information regarding their mutual patient, may also improve GPs’ referral rates.

On a higher organizational level, there is a need to find ways to provide GPs more time and skills for discussing behavioural issues with their patients. Integrating SCC into the patient journey within the health system, with a clear pathway of each health providers’ role, and better communication between the different health providers, might reduce ineffective repetition, discordant health messages, and wasted time. GPs need to receive specific training to feel confident to provide brief behavioural counselling. The frustration the GPs expressed that simply providing information on the harms of smoking does not lead to behaviour change, suggests that training needs to focus on using specific behaviour change techniques which have been shown to be effective (for example how to *facilitate goal setting*) [40, 41].

The specific barriers mentioned by GPs working with Aboriginal women, coupled with the higher smoking rates among this population, warrants separate consideration. Currently, the Australian federal government ‘closing the gap’ strategy [42], and as part of this, the “Tackling Indigenous Smoking” program is being implemented. The “Yarning about Quitting” resources were developed specifically to improve health providers’ confidence in “how” to have a culturally appropriate conversation on smoking with pregnant Aboriginal women [43]. Data on the effectiveness of these initiatives are needed. Further cost-free treatment options that are culturally appropriate need to be explored, including providing the services with free oral NRT in addition to the NRT patch.

## Conclusions

Australian GPs in this study reported a lack of knowledge and communication skills for treating pregnant women who smoke. Focusing their time on providing information on the harms of smoking, while not offering and discussing treatment options, and providing support for smoking cessation with all pregnant patients who smoke, may be contributing to the low cessation rates, and pessimism. Specific training explicitly showing ‘how to have this conversation’, including training on specific effective behaviour change techniques, with clear detailed clinical protocols on using NRT during pregnancy, may help GPs to better support their pregnant patients in their smoking cessation journey. GPs treating Aboriginal pregnant women who smoke face additional barriers that need to be addressed, from multiple levels, including policy and community levels.

## Additional files

Additional file 1: Interview Guide. A description of the topics and questions covered as part of the interview with the participants. (PDF 95 kb)

Additional file 2: **Table S1.** Self-Reported Provision of Smoking Cessation Care compared to the overall National Survey Sample, n (%). This tables compares the self-reported provision of specific smoking cessation care components between the total national survey sample, and the current study participants. **Table S2.** Barriers and Enablers to Provision of Smoking Cessation Care to Pregnant Smokers, compared to the overall National Survey Sample, n (%). (PDF 127 kb)

## Abbreviations

COM-B model: Capability, Opportunity, Motivation – Behaviour
GPs: General Practitioners
NRT: Nicotine Replacement Therapy
RACGP: The Royal Australian Collage of General Practitioners
SCC: Smoking Cessation Care
TDF: Theoretical Domains Framework
