# Supplementary material for: Overcoming Challenges to Treating Tobacco use During Pregnancy - A Qualitative study of Australian General Practitioners Barriers
Source: BMC Pregnancy Childbirth. 2019 Feb 7;19:61. doi: 10.1186/s12884-019-2208-8 (PMC6367814; doi:10.1186/s12884-019-2208-8)
Supplement: Supplementary file 1 — Interview Guide. A description of the topics and questions covered as part of the interview with the participants. (PDF 95 kb) [file 12884_2019_2208_MOESM1_ESM.pdf]

Additional file 1: Interview Guide

Topics that should be covered (and an example of a question that can be used if not already covered)

a. **Usual approach**
   i. What would you say is your usual approach to a pregnant woman who smokes?
   ii. In your experience, what have been the outcomes from your management of smoking in pregnant women?

b. **Enablers and Facilitators - General**
   iii. How do you feel about improving your management of smoking in pregnant women?
   iv. In your opinion, what could help you improve your management of smoking in pregnant women?
   v. What would help you remember to discuss this with pregnant women?

c. **Knowledge**
   vi. What are your thoughts on your knowledge to address smoking properly in pregnant women?
   vii. What would be the preferred way for you to improve your knowledge on this topic? What would be the most effective way for you?

d. **Time**
   viii. What has been your experience concerning the time frame available to address smoking properly in pregnant women?
   ix. How much time in your experience is needed for this issue? What do you think could help you incorporate this into your timeframe?

e. **Optimism**
   x. Do you feel optimistic/pessimistic about your management of smoking in pregnant women? Could you describe why that is? What would help you feel more optimistic?

f. **Confidence**
   xi. How would you describe your confidence on management of smoking in pregnant women? What would help you feel more confident?

g. **NRT**
   xii. What has been your experience with prescribing Nicotine Replacement Therapy (NRT) in pregnant women who smoke? What do you think would help you subscribe NRT to pregnant women who smoke?

h. Referral

xiii. Could you tell me a little about your experience with referring women to cessation support (such as the quit-line or a local smoking cessation group)?

xiv. What would help you to routinely refer pregnant women to cessation support?

i. Follow up

xv. What has been your experience with following up on women in regard to their smoking?

xvi. What would facilitate you to follow up?

j. Discussing the psychosocial context

xvii. How do you feel about discussing with pregnant women the psychosocial context of smoking?

xviii. What can help you with this?

k. Subgroups

xix. Describe your experience with any subgroups of pregnant women for whom there may be additional challenges to treatment?
