# Supplementary material for: Overcoming Challenges to Treating Tobacco use During Pregnancy - A Qualitative study of Australian General Practitioners Barriers
Source: BMC Pregnancy Childbirth. 2019 Feb 7;19:61. doi: 10.1186/s12884-019-2208-8 (PMC6367814; doi:10.1186/s12884-019-2208-8)
Supplement: Supplementary file 2 — Table S1. Self-Reported Provision of Smoking Cessation Care compared to the overall National Survey Sample, n (%). This tables compares the self-reported provision of specific smoking cessation care components between the total national survey sample, and the current study participants. Table S2. Barriers and Enablers to Provision of Smoking Cessation Care to Pregnant Smokers, compared to the overall National Survey Sample, n (%). (PDF 127 kb) [file 12884_2019_2208_MOESM2_ESM.pdf]

Additional file 2

Table 1: Self-Reported Provision of Smoking Cessation Care compared to the Overall National Survey Sample, n(%)

**Smoking Cessation Care Component - Proportion reporting 'Often/Always' performing each component**	**GPs participating in the qualitative interviews (n=16)**	**GPs and Obstetricians participating in the national sample (n=378)**
Ask about smoking status	16 (100%)	288 (77.2%)
Give brief advise to quit if smoking	16 (100%)	275 (74.7%)
Assess nicotine dependence	14 (87.5%)	89 (24.1%)
Provide Cessation support to smokers (Assist)	15 (92.8%)	124 (33.5%)
Follow-up within 2 weeks (Arrange)	6 (37.5%)	26 (7%)
Prescribe/recommend NRT to assist quitting	9 (56.3%)	40 (10.8%)
Discuss the psychosocial context of smoking	13 (81.3%)	82 (22.2%)
Refer to Quit line/specialist service	8 (50%)	95 (26.8%)
Involve family members in counselling/tobacco management	4 (25%)	15 (4.1%)

Table 2: Barriers and Enablers to Provision of Smoking Cessation Care to Pregnant Smokers, compared to the Overall National Survey Sample, n(%)

**TDF domains –percentage answering 'Agree/Strongly Agree' n(%)**	**GPs participating in the qualitative interviews (n=16)**	**GPs and Obstetricians participating in the national sample (n=378)**
I am confident that I can counsel women about their smoking during pregnancy	15 (93.8%)	299 (80.8%)
I am confident that I can prescribe NRT for pregnant smokers	13 (81.3%)	201 (54.5%)
I am optimistic my intervention for smoking during pregnancy is likely to be effective	6 (37.5%)	129 (35.1%)
Raising the issue of smoking with a client during pregnancy will benefit our relationship	9 (56.3%)	232 (65.2%)
Addressing smoking during pregnancy is a high priority	16 (100%)	351 (98.3%)
I am comfortable raising the issue of smoking with a pregnant woman	15 (93.8%)	338 (94.9%)
In my workplace, it is routine to help pregnant women to quit smoking during pregnancy	15 (93.8%)	271 (76.1%)
I have sufficient time to help pregnant women to quit smoking	10 (62.5%)	146 (41.1%)
I have sufficient resources to help pregnant women to quit smoking	9 (56.3%)	169 (47.5%)
